# Interventional therapy for human breast cancer in nude mice with ^131^I gelatin microspheres (^131^I-GMSs) following intratumoral injection

**DOI:** 10.1186/1748-717X-9-144

**Published:** 2014-06-23

**Authors:** Chuan-Chao Li, Jun-Lin Chi, Yu Ma, Jian-Hong Li, Chuan-Qin Xia, Lin Li, Zhuo Chen, Xiao-Li Chen

**Affiliations:** 1 Department of General Surgery, West China Hospital of Sichuan University, Chengdu (610041), China; 2 Department of Thyroid and Breast Surgery, West China Hospital of Sichuan University Chengdu (610041), China; 3 Department of Nuclear Medicine and The National Key Discipline of Medical Imaging and Nuclear Medicine, West China Hospital of Sichuan University, Chengdu (610041), China; 4 College of Chemistry, Sichuan University, Chengdu (610041), China; 5 Department of Nuclear Medicine and The National Key Discipline of Medical Imaging and Nuclear Medicine, West China Hospital of Sichuan University, Chengdu (610041), China; 6 Regeneration Medicine Research Center, West China Hospital of Sichuan University, Chengdu (610041), China

**Keywords:** ^131^I, Gelatin microspheres, Breast neoplasms, Intratumoral injection, Treatment outcome, Biodistribution

## Abstract

**Introduction:**

The aim of this study was to investigate the effects of ^131^I gelatin microspheres (^131^I-GMS) on human breast cancer cells (MCF-7) in nude mice and the biodistribution of ^131^I-GMSs following intratumoral injections.

**Methods:**

A total of 20 tumor-bearing mice were divided into a treatment group and control group and received intratumoral injections of 2.5 mci ^131^I-GMSs and nonradioactive GMSs, respectively. Tumor size was measured once per week. Another 16 mice received intratumoral injections of 0.4 mci ^131^I-GMSs and were subjected to single photon emission computed tomography (SPECT) scans and tissue radioactivity concentration measurements on day 1, 4, 8 and 16 postinjection. The 20 tumor-bearing mice received intratumoral injections of 0.4 mci [^131^I] sodium iodide solution and were subjected to SPECT scans and intratumoral radioactivity measurements at 1, 6, 24, 48 and 72 h postinjection. The tumors were collected for histological examination.

**Results:**

The average tumor volume in the ^131^I-GMSs group on post-treatment day 21 decreased to 86.82 ± 63.6%, while it increased to 893.37 ± 158.12% in the control group (*P* < 0.01 vs. the ^131^I-GMSs group). ^131^I-GMSs provided much higher intratumoral retention of radioactivity, resulting in 19.93 ± 5.24% of the injected radioactivity after 16 days, whereas the control group retained only 1.83 ± 0.46% of the injected radioactivity within the tumors at 1 h postinjection.

**Conclusions:**

^131^I-GMSs suppressed the growth of MCF-7 in nude mice and provided sustained intratumoral radioactivity retention. The results suggest the potential of ^131^I-GMSs for clinical applications in radiotherapy for breast cancer.

## Introduction

Selective internal radiotherapy (SIRT), using radioactive microspheres or particles, is a rapidly developing technology in the field of minimally invasive interventional cancer therapy. The major advantage of internal radiation over external radiation is that, by directly delivering radiopharmaceuticals into tumor tissue, internal radiation can maximize the antineoplastic effects on tumor tissue, while preventing normal tissues from radiation damage [1]. In addition, tumor cells can be continuously exposed throughout the cell cycle to the radiation of radionuclides carried by the microspheres or particles. In recent years, radiolabeled microspheres and particles have been used in clinical settings or animal studies, including ^90^Y glass or resin microspheres [2,3], ^32^P glass microspheres or colloids [4,5], ^166^Ho-loaded glass microparticles [6], ^186/188^Re glass microspheres [7] and various biodegradable microspheres, such as ^166^Ho poly(L-lactic acid) microspheres(PLLA-MS) [8], and these microspheres and particles showed excellent anti-tumoral effects and a good level of safety after intraarterial or intratumoral injections. There are two forms of commercially available ^90^Y microspheres — *TheraSphere*® and *SIR-Spheres*® — which were approved by the FDA, respectively, in 2000 for use in radiation treatment for inoperable hepatocellular carcinoma and in 2002 for the treatment of colorectal cancer metastasized to the liver [9]. Studies have shown that intraarterial infusion of ^90^Y microspheres is an effective and safe alternative for treating patients with inoperable hepatocellular carcinoma and metastatic liver cancer originating from colorectal carcinoma, breast cancer and endocrine system cancers [2,3,10-13]. In addition to radio-embolization, direct intratumoral injection of radiolabeled microspheres or particles is also an effective and safe method for treating malignancies [5,14], and this treatment is especially suitable for treating solid tumors characterized by hypo-vasculature [15] or for which intraarterial interventional therapy is infeasible, such as breast cancer and prostate cancer. As we know, radioembolization is defined as the injection of radiolabeled microspheres or particles by use of percutaneous superselective catheterization of the tumor vasculature, so it is a very challenging work and it may result in severe adverse effect once the non-target vessels are embolized. Up to now, percutaneous radioembolization is only used in the internal radiotherapy of liver malignant tumors, and it is infeasible in the treatment of many other tumors, such as breast cancer and prostate cancer, in which image-guided superselective catheterization of the tumor vessels almost can’t implement.

The radioactive microspheres consist of two parts: a radionuclide and carrier. Although the application of ^90^Y microspheres in radiotherapy for malignancies has already obtained considerable success, its drawbacks are also obvious. For example, as ^90^Y is a pure beta emitter and does not produce gamma rays, the biodistribution of ^90^Y microspheres cannot be directly determined by SPECT scans [16]. In addition, the high density of glass increases the likelihood of premature intravascular settling and falling back into the gastrointestinal tract, thus resulting in radiation-related gastrointestinal side effects [17,18], and the non-biodegradability of glass or resin could hamper the repeated administration of microspheres. Therefore, biodegradable microspheres labeled with radioisotopes that simultaneously yield βemissions and γ rays have attracted increasing attention in recent years. In fact, some biodegradable microspheres have been successfully developed, such as poly (L-lactic acid) microspheres loaded with ^166^Ho or ^186/188^Re, which showed high degrees of *in vitro* and *in vivo* stability and good safety profiles [8,19,20].

We have been studying radioiodine-labeled gelatin microspheres in recent years. [^131^I] Sodium iodide (Na^131^I) oral solution is readily obtained, as it is routinely used for the treatment of hyperthyroidism and differentiated thyroid carcinoma. The major advantage of ^131^I over other radionuclides is that dissociative iodine mainly accumulates in the thyroid or is directly excreted via the kidneys [21-24] and rarely remains in other tissues, so oral administration of a single dose of more than 100 mci Na^131^I solution is relatively safe for patients with differentiated thyroid carcinoma or thyrotoxicosis, and the radiation dose to the family members of patients treated with up to 600 MBq of radioiodine is well below the recommended dose constraints if safety instructions are complied with well [25,26]. Gelatin microspheres are a type of derivative of collagen with a good level of biocompatibility, and they can be labeled with a high concentration of radioiodine. In our previous studies, we injected ^131^I labeled gelatin microspheres and ^131^I, ^125^I dual-labeled gelatin microspheres into the liver parenchyma of rabbits, and we found that the injected microspheres mainly accumulated around the injection site, and the small amount of de-labeled radioiodine did not cause severe damage to other tissues [27,28]. In the present study, we evaluated use of ^131^I-GMSs as an effective radiopharmaceutical for the treatment of transplanted human breast cancer (MCF-7) in nude mice following intratumoral injections.

## Materials and methods

### Preparation of GMSs

The preparation of ^131^I-GMSs have been previously described in detail [27]. In brief, 6 mL of gelatin solution (15 wt%) was dropwise added to 40 mL of liquid paraffin (Kelong Chemical Reagent Co. Ltd., Chengdu, China) with 0.4 mL of Span 80 (Shenyu Chemical Reagent Co. Ltd., Chongqing, China), and stirred at 600 rpm in a 50°C water bath, after 20 min minutes later, the solutions were rapidly cooled to 4°C with continuous stirring. Then cross-bonding was performed by adding 2 mL of 25% glutaraldehyde (25%, Kermel Chemical Reagent Co. Ltd., Tianjin, China) with stirring for 30 minutes. Solid gelatin microspheres were collected after rinsed in acetone (Changlian Chemical Reagent Industries, Ltd., Chengdu, China) and sieved the microspheres of size 30 to 50 microns with a stainless steel sieve. Then we labeled ^131^I by a modification of the chloramine-T method. Briefly, 50 mg of gelatin microspheres was added into 190 μL of phosphate-buffered saline (pH 7.0) for swelling in test tubes. 10 min later, 20 μL of ^131^I-sodium-iodine solution (37 GBq/mL) and 200 μL of chloramine-T solution (Bodi Chemical Reagent Co. Ltd., Tianjin, China) (20 mg/mL) were added. After mixing for 30 min with a vortex at room temperature, 200 μL of sodium pyrosulfite solution (Jiangbei Chemical Reagent Industries, Ltd., Wuhan, China) (15 mg/mL) was added to stop the reaction. Then the mixtures was centrifuged at 1000 r/min for 4 min to separate the ^131^I- gelatin microspheres. Finally, the products were washed seven times with normal saline and sterilized by ^60^Co irradiation.

### Human breast cancer xenografts in nude mice

This study was approved by the animal ethics committee of Sichuan University. Nude mice 4–5 weeks age (BABL/c) were provided by the experimental animal center of Sichuan University. The nude mice, 4–5 animals per cage, were housed in pathogen-free conditions, with sterile water and granular food *at libitum*. The air humidity and temperature were maintained at 50%-70% and 20-29°C, respectively. Human breast cancer cell line MCF-7 was purchased from China Center for Type Culture Collection (CCTCC, Wuhan, China), and the cells were cultured in RPMI-1640 medium supplemented with 10% fetal calf serum (Thermo-Fisher Biochemical Products [Beijing] Co., Ltd., Beijing, China) and were incubated at 37°C in a 5% CO_2_ incubator. After growing exponentially for 1 week, the MCF-7 cells were harvested by trypsinization and were washed with normal saline, and approximately 1 × 10^7^ MCF-7 cells were then suspended in 0.1 mL of normal saline and injected into the mammary fat pads of each female nude mouse.

### Tumor volume and body weight measurements

A total of 20 tumor-bearing nude mice were randomly divided into 2 groups and were used to evaluate the efficacy of treatment. When the tumors reached approximately 1.0 cm -1.5 cm in diameter, microspheres were suspended in 0.1 mL of glucose solution (25%) and were slowly injected into the centers of the tumors using a 1 mL syringe and a 27G needle. The treatment group (n = 10) and the control group (n = 10) were treated with 2.5 mci (92.5 MBq) ^131^I microspheres and the same amount of nonradioactive microspheres, respectively. The preparation of ^131^I-GMSs has been previously described in detail [27].

The animals were monitored for the treatment’s effects on tumor size and body weight for 7 weeks or until the animals reached the maximum allowable tumor burden. The tumor size was measured with Vernier calipers in two dimensions, and tumor volume was calculated as *v =1/2ab*^*2*^, where *a* and *b* are the largest and smallest diameters of the tumor, respectively, and *v* is the tumor volume in cubic centimeters. The therapeutic effects of ^131^I-GMSs were evaluated by comparing the tumor volume of the treatment group with that of the control group. In addition, the body weights of the animals were also measured to evaluate the radiation toxicity.

### Tissue radioactivity concentration measurements and image acquisition

Twenty nude mice were injected with 0.4 mci (14.8 MBq) ^131^I-GMSs and were used to determine the tissue biodistribution of ^131^I on days 1, 4, 8 and 16 after ^131^I-GMSs injections (4 animals for each time point). Under anesthesia by intraperitoneal injection of 0.1 ml chloral hydrate (West China Hospital), a single photon emission computed tomography (SPECT) scan was obtained, and SPECT-CT images were acquired using a gamma camera (Skylight SPECT camera, Philips Co., Ltd., Amsterdam, the Netherlands) in the Nuclear Medicine Department of West China Hospital. Then, the animals were sacrificed by cervical dislocation, and samples of the liver, lung, spleen, kidney, muscle, bone, thyroid gland and whole tumor were excised and weighed carefully. Radioactivity was measured by a γ counter (No. 262 Industry, Ltd., Xi’an, China). In addition, 1 ml of blood was collected from the orbital vein and was used for γ counting. The intratumoral retention of injected radioactivity was expressed as a percentage of the injected dose in the whole tumor; radioactivity concentrations in other organs and in serum were expressed as percentages of the injected dose per gram (% ID/g) and per milliliter, respectively.

To evaluate the role of ^131^I-GMSs in the intratumoral retention of radioactivity, another 20 tumor-bearing nude mice were used as a control group. After intratumoral injection of 0.4 mci [^131^I] sodium iodide oral solution (Na^131^I),SPECT-CT images were acquired, and the intratumoral radioactivity levels were measured at 1, 4, 24, 48 and 72 h postinjection (4 animals for each time point).

### Histological findings

Tumor samples were fixed by immersion in 10% formalin solution for 48 h, embedded in paraffin, sectioned and stained with hematoxylin and eosin (HE) for histological examination. The tumor samples at 28 days and 35 days after injection were also examined.

### Data analysis

The results of the tumor volumes are expressed as the means ± SDs and were analyzed by one-way ANOVA using SPSS software, version 19.0, and the level of significance was set at a *P* value < 0.05.

## Results

### Scanning electron microscopic images of ^131^I-GMSs

**Figure 1:**
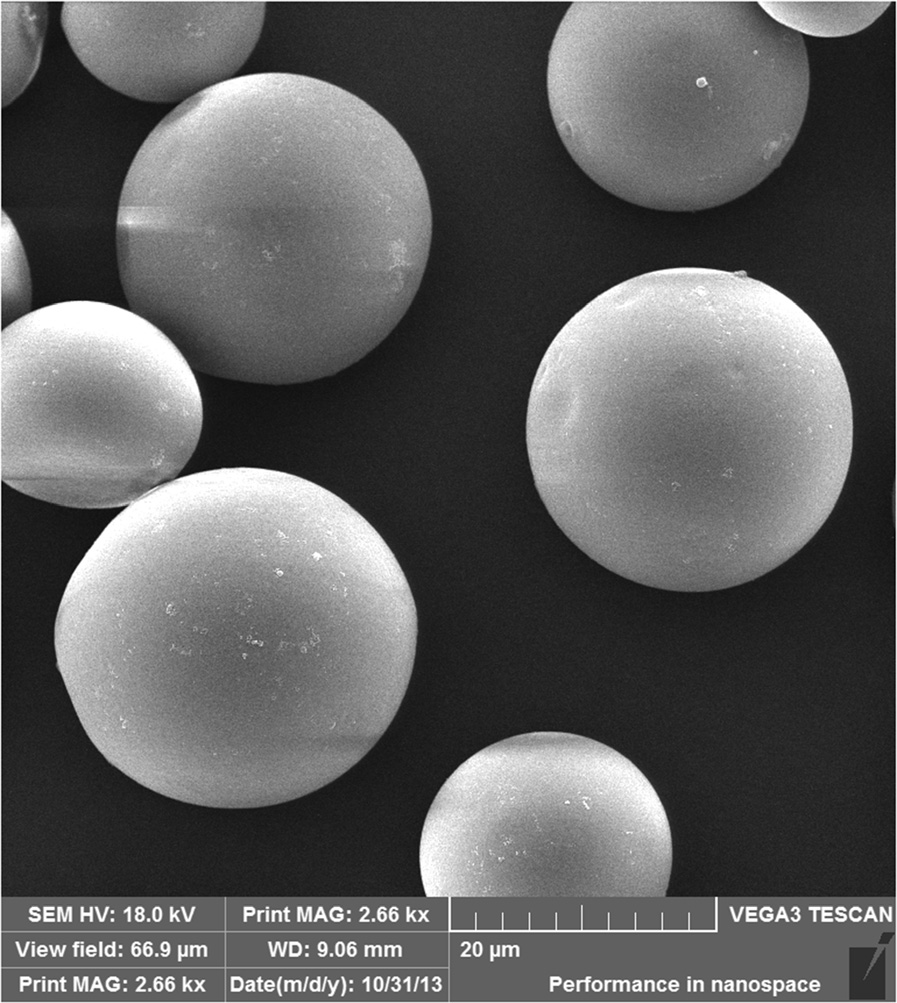
**Scanning electron microscopic images of **^**131**^**I gelatin microspheres.**

Figure 1 shows the scanning electron microscopic images of ^131^I-GMSs. The microspheres were washed with normal saline 7 times after labeling to minimize physical absorption, and the final labeling rate of ^131^I was 57.5 ± 2.62%. The microspheres were uniform in shape, with a diameter of 30–50 μm and good divergence.

### Tumor volumes in the ^131^I-GMSs treatment group and control group

**Figure 2:**
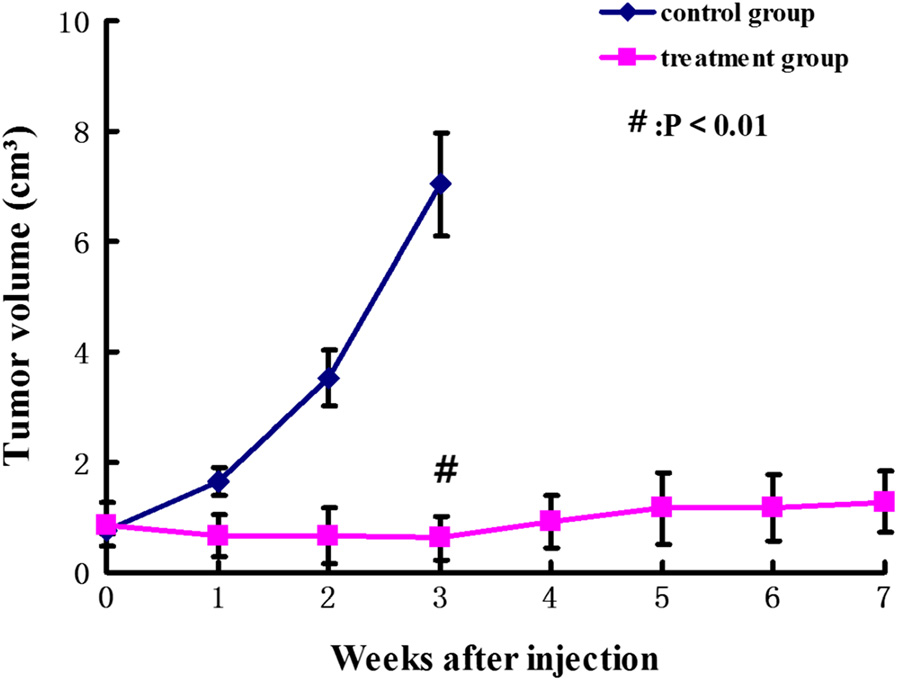
**Tumor volumetric assessment of the effect of **^**131**^**I-GMSs on MCF-7 xenografts in nude mice.** The points represent means, and the bars represent standard errors. Comparisons between control and ^131^I-GMSs group were analyzed by one way ANOVA, and a P value < 0.05 is regarded as significance. It is obvious that intratumoral injection of 2.5 mci ^131^I-GMSs significantly suppressed the growth of tumors compared with control group, **#**: *P* <0.01.

Figure 2 shows the changes in tumor volumes in the ^131^I-GMSs treatment group and the control group. On the day of microsphere injection, the average tumor volumes in the ^131^I-GMSs treatment group and the control group were 0.87 ± 0.39 cm^3^ and 0.79 ± 0.06 cm^3^, respectively, and the difference was not significant (*P* > 0.05). On day 21 postinjection, the average tumor volume of the treatment group decreased to 0.63 ± 0.39 cm^3^, which was 86.82 ± 63.6% of the initial volume (*P* < 0.01 compared to the control group), whereas the average tumor volume in the control group increased to 7.03 ± 0.95 cm^3^, which was 893.37 ± 158.12% of the pre-treatment volume. The rates of tumor growth in the ^131^I-GMSs treatment group and the control group over this period were -0.01 ± 0.02 cm^3^/d and 0.30 ± 0.05 cm^3^/d, respectively. It is obvious that intratumoral injection of 2.5 mci ^131^I-GMSs statistically significantly suppressed the growth of tumors compared with the control group. At the end of the 7^th^ week (the endpoint of the study), the average tumor volume of the treatment group was 1.27 ± 0.55 cm^3^, which was 186.2 ± 55.2% of their initial volume. The control group animals were sacrificed on day 21 because of excessive tumor burden.

### Biodistribution of ^131^I

The radioactive concentrations after injection of ^131^I-GMSs are summarized in Table 1. The radioactivity in the tumors was much higher than in any other tissue. The highest radioactivity concentration outside the tumors was found in the lung, which had 2.67% of the injected radioactivity on the 1^st^ day and 1.89% on the 4^th^ day, but this level rapidly decreased to 0.34% by the 8^th^ day. The radioactivity concentrations in other tissues, such as the kidney, liver, spleen, bone, muscle, serum, and even thyroid gland, were quite low throughout the observation period. The intratumoral retention of injected radioactivity (% ID) after injection of ^131^I-GMSs was 43.29 ± 5.27%, 29.28 ± 3.72%, 24.71 ± 7.28% and 19.93 ± 5.24% at 1, 4, 8 and 16 days, respectively, whereas the intratumoral retention of injected radioactivity (% ID) after injection of Na^131^I solution was only 1.83 ± 0.46%, 0.60 ± 0.29%, 0.45 ± 0.07%,0.14 ± 0.02% and 0.06 ± 0.01% at 1, 6, 24, 48 and 72 h after injection, respectively (Table 2).

**Table 1:** **Biodistribution after intratumoral injections of **^**131**^**I-GMSs (%, mean ± SD)**

**Time**	**Liver**	**Thyroid**	**Lung**	**Kidney**	**Spleen**	**Bone**	**Muscle**	**Serum**	**Tumor**
1 d	0.09 ± 0.03	0.33 ± 0.20	2.67 ± 0.42	0.57 ± 0.34	0.1 ± 0.04	0.51 ± 0.10	0.12 ± 0.07	0.16 ± 0.06	43.29 ± 5.27
4 d	0.07 ± 0.02	0.12 ± 0.05	1.89 ± 0.58	0.69 ± 0.30	0.04 ± 0.01	0.05 ± 0.02	0.02 ± 0.01	0.09 ± 0.01	29.28 ± 3.32
8 d	0.01 ± 0.00	0.02 ± 0.01	0.34 ± 0.25	0.05 ± 0.03	0.06 ± 0.01	0.11 ± 0.03	0.02 ± 0.01	0.04 ± 0.01	24.71 ± 7.28
16 d	< 0.01	0.02 ± 0.00	0.10 ± 0.06	0.02 ± 0.01	0.01 ± 0.00	0.09 ± 0.01	0.02 ± 0.01	0.05 ± 0.01	19.93 ± 5.24

The biodistribution of radioactivity after intratumoral injections of ^131^I-GMSs. The intratumoral retention of injected radioactivity into the tumors was expressed as the percentage of the injected dose in the whole tumor; tissue concentrations of other organs were expressed as the percentage of the injected dose per gram (% ID)/g, and the radioactivity in the serum was expressed as the percentage of the injected dose per milliliter.

**Table 2:** The comparisons of the intratumoral retention of radioactivity (%, mean ± SD)

^**131**^**I-GMSs**	**1d**	**4d**	**8d**	**16d**	
	43.29 ± 5.27	29.28 ± 3.72	24.71 ± 7.28	19.93 ± 5.24	
Na^131^I solution	1 h	6 h	24 h	48 h	72 h
	1.83 ± 0.46	0.60 ± 0.29	0.45 ± 0.07	0.14 ± 0.02	0.06 ± 0.01

The comparison of the intratumoral retention of radioactivity after intratumoral injections of ^131^I-GMSs and Na^131^I solution.^131^I-GMSs provided sustained intratumoral retention of injected radioactivity, maintaining 19.93 ± 5.24% of the injected radioactivity within the tumors after 16 days. In contrast, radioactivity was cleared rapidly from the tumors after injection of Na^131^I solution. By 1 h, the Na^131^I solution–treated group retained only 1.83 ± 0.46% of the injected radioactivity within the tumors.

### SPECT-CT fusion images

**Figure 3:**
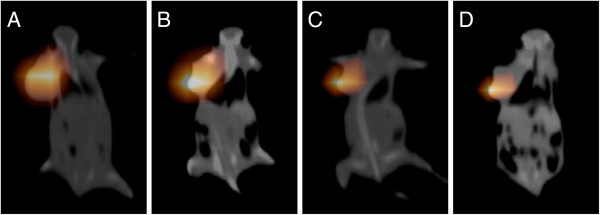
**The results of SPECT imaging at 1 (A), 4 (B), 8 (C) and 16 days (D) after intratumoral administration of **^**131**^**I-GMSs.** The injected radioactivity concentrated in the tumors, and no accumulation of radioiodine was noted by SPECT scan in other tissues.

**Figure 4:**
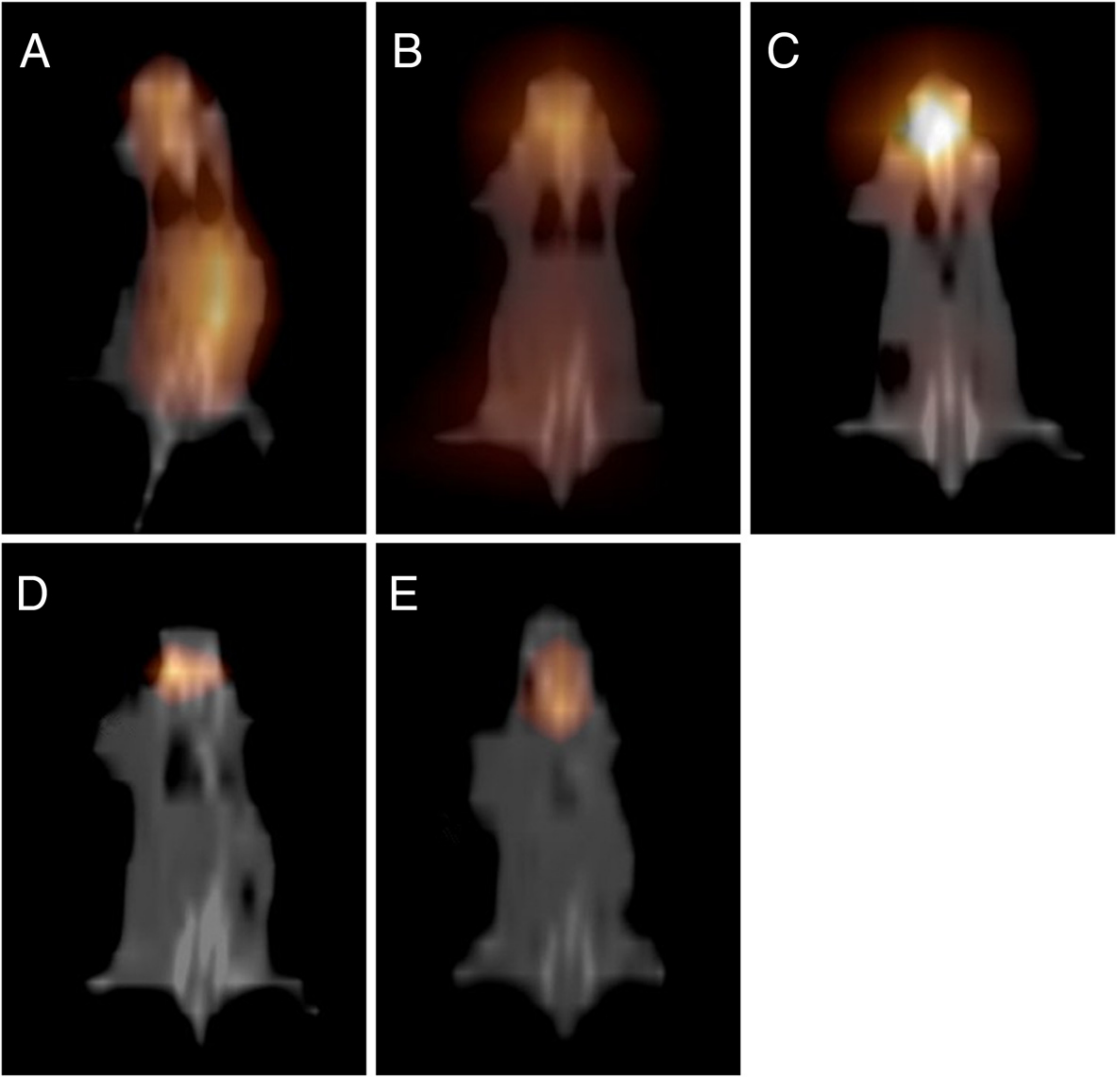
**The results of SPECT imaging at 1 (A), 6 (B), 24 (C), 48 (D) and 72 h (E) after the administration of [**^**131**^**I] sodium iodide oral solution.**

A SPECT scan was obtained on day 1, 4, 8 and 16 for the ^131^I-GMSs group and at 1, 6, 24, 48 and 72 h for the Na^131^I solution group, respectively. SPECT imaging showed that the radioisotopes were concentrated in the tumor for the whole observation period, and no accumulation of nuclides was noted in the other tissues, including the thyroid gland and the urinary system (Figure 3A-D). However, SPECT imaging showed diffused biodistribution of the injected radioactivity throughout the whole body 1 h after intratumoral injection of Na^131^I solution (Figure 4A), followed by gradual accumulation of ^131^I in the thyroid area and decreases in radioactivity in other tissues (Figure 4B,C). At 48 h after injection, the radioactivity was mainly concentrated in thyroid area, and no radioactivity was noted in any other tissue (Figure 4D,E). The intratumoral radioactivity was quite low throughout the observation period in this group (Figure 4A-E).

### Pathological findings

**Figure 5:**
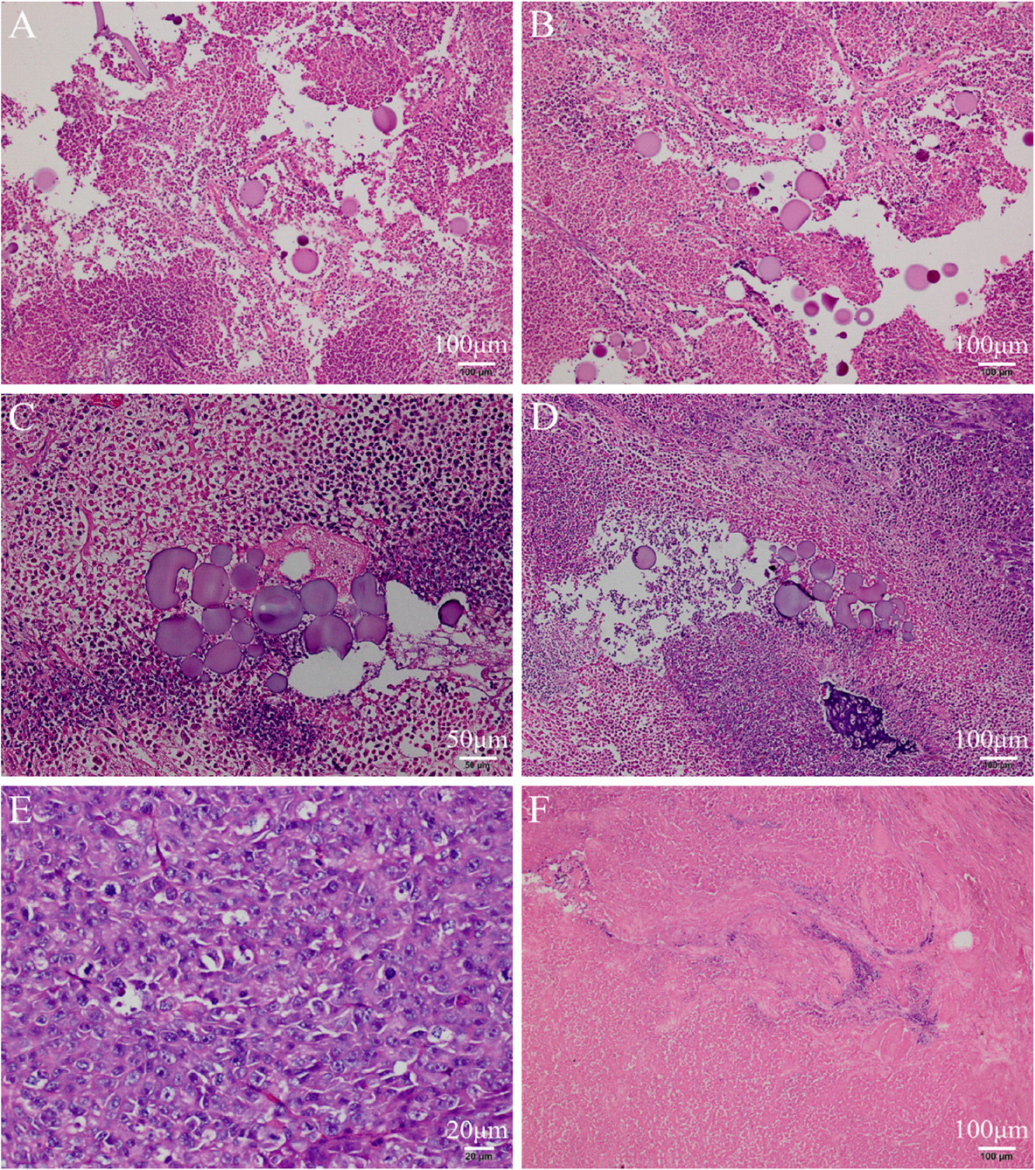
**Pathological examination at 1 (A), 16 (B), 28(C) and 35(D) days after the injection of 0.4 mci **^**131**^**I gelatin microspheres, at 21 days after administration of unlabeled microspheres (E) and at 49 days after administration of 2.5 mci **^**131**^**I-GMSs (F).** (HE stain; original magnification, ×100 in **A**, **B, D** and F; ×200 in C and × 400 in E).

Pathological examination showed that the injected microspheres concentrated around the injection site, with intact spheric shape at 1 day (Figure 5A). The microspheres were degraded gradually after injection (Figure 5A,B,C,D,E and F). At 35 days after injection, microspheres degraded to irregular shape, with some parts of microspheres missing. However, some microspheres still retained their complete morphology (Figure 5D). In treatment group, there were no visible microspheres at 49 days after injection of ^131^I-GMSs (Figure 5F).

### Body weight

**Figure 6:**
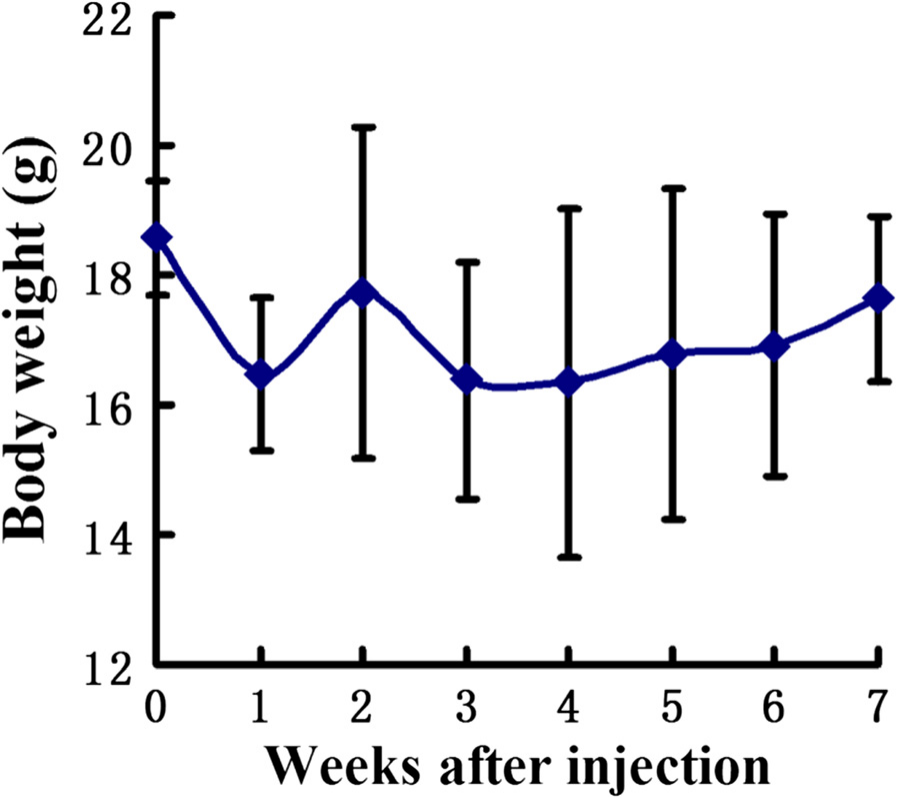
**The average body weight in **^**131**^**I-GMSs group.**

The ^131^I-GMSs group showed mild body weight loss of 12.16 ± 10.1% from postinjection to day 21, but they gradually gained weight thereafter (Figure 6).

## Discussion

Breast cancer is the second most common non-skin cancer in women worldwide, with an incidence of 10.4%, and it has the highest incidence among all cancer types in women in the USA, with one in every 8–10 women being affected during her lifetime [29]. Prospective trials have clearly demonstrated the efficacy of breast conservation therapy (BCT) for early breast cancer, showing that it offers equivalent local control and long-term survival compared with mastectomy [30-33]. As an indispensable part of BCT, radiation therapy has a significant role in the treatment of breast cancer [34,35]. However, the rationality of conventional whole breast irradiation (WBI) has gradually been questioned. On the one hand, local recurrence tends to occurs at, or in proximity to, the tumor bed, and other sites of ipsilateral breast recurrence occur rarely, in 3%-4% of all cases [36]; on the other hand, radiation therapy limited to the region of the tumor bed (APBI) produced long-term local control and survival rates comparable to those from WBI in selected low-risk patients [37]. Accelerated partial breast irradiation (APBI) is an approach that treats only the tumor bed plus a 1- to 2 -cm margin, rather than the whole breast, thus irradiating a smaller volume, giving a higher radiation dose per fraction to the tumor bed. This process shortens the treatment time significantly [38].

Interstitial breast brachytherapy is an APBI technique that has been practiced for more than 20 years and that has the most extensive follow-up [39]. Many prospective studies have reported low local recurrence with brachytherapy at 5 and 10 years, comparable to WBI [37,40-42]. For example, Polgár et al. reported their experience with APBI, providing the longest follow-up in the literature for the HDR multi-catheter techniques, and the 5- and 12-year local recurrence rates were only 4.4% and 9.3%, respectively [42]. However, the implementation of catheter insertion in multi-catheter interstitial brachytherapy requires a high level of skill on the part of the operator and the support of sophisticated imaging techniques. In contrast, direct interstitial injection of radiopharmaceuticals is relatively easy to implement. As ^131^I gelatin microspheres are a biodegradable material and can be injected repeatedly, they could have potential clinical value in treating the tumor bed after breast conservation surgery. Therefore, in this study, we evaluated the effectiveness of ^131^I-GMSs in MCF-7 tumors after direct injection into these tumors in nude mice. Additionally, we studied the biodistribution of dissociative ^131^I following the degradation of the microspheres.

Long-term intratumoral retention of radiation sources is essential for effective brachytherapy of malignancies, as it maximizes the antineoplastic effects on tumor tissue for longer periods. In the present study, the diameter of the injected microspheres (30–50 μm) (Figure 1) was significantly larger than the endothelial gap of vasculature in tumor tissue, which effectively prevented escaping of the microspheres from the tumors. Our research showed that ^131^I-GMSs provided sustained intratumoral radioactivity retention. The intratumoral radioactivity level in the ^131^I-GMSs group was 19.93 ± 5.24% of the injected dose on day 16 postinjection but only 1.83 ± 0.46% in the control group at 1 h after injection (Table 2). This sustained intratumoral retention of radioactivity after intratumoral injection of 2.5 mci ^131^I-GMSs resulted in a significantly slower tumor growth rate. On day 21, the average tumor volume in the control group was 893.37 ± 158.12% of the pre-treatment volume, whereas the tumor volume in the treatment group was only 186.2 ± 55.2% of that before injection at the end of 7^th^ week (Figure 2).

The safety profile of internal radiotherapy is largely dependent of the *in vivo* biodistribution of the radioactivity. As the most straightforward method of bringing radionuclides into tumors, direct intratumoral injection of radiopharmaceuticals can maximize radiation damage to the tumor tissue, while sparing toxicity to the critical structures that might be nearby or superficial to the target area [43]. In our study, the intratumoral radioactivity level was significantly higher than that in any other tissue throughout the study (Table 1). The lung was the only tissue, other than the tumor, that showed relatively high radioactivity concentrations, which were 2.67 ± 0.42% and 1.89 ± 0.58% of the injected dose per gram (% ID/g) at 1 day and 4 days postinjection, respectively, but this level decreased rapidly to 0.34 ± 0.25% at 8 days postinjection.

As gelatin microspheres are a type of biodegradable carrier of radionuclides, the biodistribution of de-labeled ^131^I should be evaluated dynamically, along with the degradation of the microspheres. The biodistribution study showed that the concentrations of radioactivity in the liver, spleen, kidney, bone, muscle, and even thyroid gland were quite low throughout the entire observation period (Table 2), suggesting that gradual degradation did not cause abundant de-labeling of ^131^I from the microspheres. In recent years, studies have shown the potential of gelatin microspheres used as carriers of drugs or cytokines. The degradation period of gelatin *in vivo* can be controlled by changing its degree of cross-linking [44,45], thus enabling the sustained and controlled release of drugs and cytokines [46-48]. When gelatin microspheres are used as carriers of radionuclides in brachytherapy for malignancies, it is worth noting that the degradation period should be regulated according to the half-lives of the radionuclides, thus preventing release of the radionuclides in large quantities from the microspheres due to premature degradation of the microspheres.

^131^I simultaneously yields γ-rays (1%, 0.364 Mev) and β-emissions (99%, 0.606 Mev). Although the low-energy γ-rays are almost negligible for the treatment of tumors, they might be useful in determining the biodistribution of microspheres. Studies have demonstrated the potential of ^166^Ho-PLLA-MS, another γray emitter, for predicting the biodistribution of the same microspheres. The distribution of scout doses and treatment doses of ^166^Ho-PLLA-MS were nearly identical, suggesting that a scout dose of ^166^Ho-PLLA-MS pretreatment could be used to predict the biodistribution of a treatment dose [49]. It is expected that if used in the radioembolization of liver malignancies, an intraarterial injection of a small dose of ^131^I-GMSs pretreatment could also be used to predict the biodistribution of a treatment dose of ^131^I-GMSs, which would be helpful in recognizing patients contraindicated for radio-embolization due to elevated lung shunting fractions [50].

The antitumoral effect and the process of the degradation of the ^131^I microspheres were confirmed by a histological examination. At 21 days postinjection in the group received non-radioactive microspheres, the tumors showed hyper-cellular neoplastic cells, with significant increased nuclear-cytoplasmic ratio, and no signs of necrosis were observed (Figure 5E). 7 weeks after injection of ^131^I-GMSs in treatment group, the tumor tissue showed large area of necrosis, with only very small amounts of residual tumor cells (Figure 5F). At 35 days after injection, there were still large number of microspheres within the tumors (Figure 5D). In our previous studies, the microspheres which were injected into the parenchyma of liver of rabbits could still be observed by histological method even 32 days( 4 half-lives) after injection [27,28]. Perhaps much more effort is needed to retard the degradation of the microspheres, thus minimizing the de-labeling of radioiodine.

In conclusion, our research shows that intratumoral injection of ^131^I labeled gelatin microspheres significantly suppressed tumor growth in a nude mouse model of human breast cancer. The injected radioactivity mainly accumulated within the tumors, and along with the degradation of ^131^I-GMSs, the radioactivity concentration in all other tissues was quite low throughout the study. Although a lot more work remains to be done, these results suggest the potential clinical value of intratumoral injections of ^131^I-GMSs in the treatment of tumor beds after breast conservation surgery.

## Abbreviations

GMSs: Gelatin microspheres; SIRT: Selective internal radiotherapy; SPECT: Single photon emission computed tomography; CT: Computed tomography.

## Competing interest

The authors declare that they have no competing interests.

## Authors’ contributions

CXL, LL, XCQ and CJL designed the research; LCC, CJL, and LJH performed the research; LCC, CJL and CXL analyzed the data and wrote the paper. LCC and CJL are the co-first author. All authors read and approved the final manuscript.

## Financial support

This research was supported by the Natural Science Pillar Program of Sichuan Province, China, No.2013JY0009.
